# Genome-wide profiling of retroviral DNA integration and its effect on clinical pre-infusion CAR T-cell products

**DOI:** 10.1186/s12967-022-03729-5

**Published:** 2022-11-08

**Authors:** Lipei Shao, Rongye Shi, Yingdong Zhao, Hui Liu, Alexander Lu, Jinxia Ma, Yihua Cai, Tatyana Fuksenko, Alejandra Pelayo, Nirali N. Shah, James N. Kochenderfer, Scott M. Norberg, Christian Hinrichs, Steven L. Highfill, Robert P Somerville, Sandhya R. Panch, Ping Jin, David F. Stroncek

**Affiliations:** 1 Department of Transfusion Medicine, Center for Cellular Engineering, NIH Clinical Center, Bethesda, MD 20892 USA; 2 Division of Cancer Treatment and Diagnosis, Biometric Research Program, National Cancer Institute, Rockville, MD 90033 USA; 3 Pediatric Oncology Branch, Center for Cancer Research, NCI, NIH, Bethesda, MD 20892 USA; 4 Surgery Branch, Center for Cancer Research, NCI, NIH, Bethesda, MD 20892 USA; 5 Genitourinary Malignancies Branch Cellular Therapy program, Cancer Immunotherapy Program, NCI, NIH, Bethesda, MD 20892 USA

## Abstract

**Background:**

Clinical CAR T-cell therapy using integrating vector systems represents a promising approach for the treatment of hematological malignancies. Lentiviral and γ-retroviral vectors are the most commonly used vectors in the manufacturing process. However, the integration pattern of these viral vectors and subsequent effect on CAR T-cell products is still unclear.

**Methods:**

We used a modified viral integration sites analysis (VISA) pipeline to evaluate viral integration events around the whole genome in pre-infusion CAR T-cell products. We compared the differences of integration pattern between lentiviral and γ-retroviral products. We also explored whether the integration sites correlated with clinical outcomes.

**Results:**

We found that γ-retroviral vectors were more likely to insert than lentiviral vectors into promoter, untranslated, and exon regions, while lentiviral vector integration sites were more likely to occur in intron and intergenic regions. Some integration events affected gene expression at the transcriptional and post-transcriptional level. Moreover, γ-retroviral vectors showed a stronger impact on the host transcriptome. Analysis of individuals with different clinical outcomes revealed genes with differential enrichment of integration events. These genes may affect biological functions by interrupting amino acid sequences and generating abnormal proteins, instead of by affecting mRNA expression. These results suggest that vector integration is associated with CAR T-cell efficacy and clinical responses.

**Conclusion:**

We found differences in integration patterns, insertion hotspots and effects on gene expression vary between lentiviral and γ-retroviral vectors used in CAR T-cell products and established a foundation upon which we can conduct further analyses.

**Supplementary Information:**

The online version contains supplementary material available at 10.1186/s12967-022-03729-5.

## Introduction

Chimeric antigen receptor (CAR)-engineered T-cells have become an important cancer immunotherapy and the clinical application of these cells for the treatment of hematological cancers is increasing rapidly [1–3]. For most CAR T-cell therapies, autologous T-cells are genetically modified to express a specific chimeric antigen receptor, which have high specificity and affinity for antigens expressed on the surface of target cells [1–3]. Currently, the clinical production of CAR T-cells relies to a great extent on T-cell transduction using viral vectors, mainly γ-retroviral and lentiviral vectors, to deliver the engineered receptors of interest. Both γ-retroviruses and lentiviruses are members of the retroviridae family, which are characterized by their ability to retrotranscribe RNA genome into a cDNA copy and stably integrate into the host cell genome [4, 5]. However, most of these integration events occur randomly. In some cases of CAR T-cell therapy, vector integration has resulted in a growth advantage which led to clonal CAR T-cell expansion and dominance [6, 7]. CAR T-cells with viral vector integration events in the exons of TET2 and CBL have resulted in clonal expansion and complete disease remission [6, 7]. While CAR T-cell therapy has proven to be relatively safe regarding long-term and unanticipated impacts of vector integration [8, 9], serious adverse effects associated with vector integration have been observed in human gene-therapies targeting immune deficiencies. In clinical trials for X-linked Severe Combined Immunodeficiency (SCID-X), for instance, a therapeutic retroviral vector integrated near the LMO-2 proto-oncogene locus and caused leukemia-like illness [10, 11]. While such leukemia-like illnesses have not occurred with clinical CAR T-cell therapy using standard transduction methodologies, the potential effects of these insertion sites on CAR T-cell safety and potency is still unclear. Thus, it is essential to comprehensively explore the precise viral vector integration sites and evaluate their potential effects on CAR T-cell safety and potency.

Next-generation sequencing and various cutting-edge biomolecular techniques have made it possible to identify viral vector integration sites across the entire genome. Several methods have been developed to investigate viral insertion events [12–18]. These studies have reported that integration may occur in different chromosomes and regions of the human genome. Furthermore, vector integration preferentially occurs at fragile sites, transcriptionally active regions and those recurrently involved in translocation events [19–23]. However, most of these studies have been limited to human immunodeficiency virus (HIV), human papillomavirus (HPV) or Murine leukemia virus (MLV) infected cells and little is known about the characteristics of viral integration events in CAR T-cells. In addition, the effects of viral integration events on the transcriptome of CAR T-cells and their association effects on clinical outcomes are not known.

Here, we explored the insertional sites of γ-retroviral and lentiviral vectors in clinical CAR T-cell products using a vector integration sites analysis (VISA) pipeline, which was modified from previously reported workflows [12–15, 17, 18]. Combined with RNA-seq data and clinical outcomes, we further explored the insertional effects on gene expression and their association with clinical outcomes.

## Materials and methods

### Collection of CAR T-cell products

A total of 75 CAR T-cell and 6 TCR T-cell products were analyzed. 57 products were manufactured with lentiviral vectors: CD22-CAR T-cells (n = 41) [3], CD19/CD22 CAR T-cells (n = 13), CD30-CAR T-cells (n = 2) [24] and FGFR4-CAR T-cells (n = 1). 24 products were manufactured with γ-retroviral vectors: BCMA-CAR T-cells (n = 11) [25], SLAMF7-CAR T-cells (n = 6) [26], CD19-CAR T-cells (n = 1) and E7-TCR T-cells (n = 6) [27]. All products (paired with non-transduced cells, which were cultured in the same manner) were sampled before they were infused into the patient. A cell pellet from the CAR T-cell product was collected and saved for DNA and RNA extraction. All subjects provided written consent.

### DNA extraction and vector integration site analysis

Genomic DNA was extracted from pre-infusion CAR T-cell products according to Qiagen DNeasy Blood and Tissue Kit (Cat#69,506, Qiagen) and. Purity and concentration were measured using Nanodrop spectrophotometer (Thermo Fisher Scientific). 2.5 µg gDNA was used to construct pools of adaptor-ligated hDNA-vDNA fragment libraries according to the manual of Retro-X/Lenti-X Integration Site Analysis Kit (Cat#631,467, Cat#631,263, Takara). Briefly, genomic DNA from CAR T-cell products was random sheared with restriction enzyme *DraІ*, end-paired and ligated with GenomeWalker adaptors (supplied from Takara). hDNA-vDNA fragments were amplified with primers specific to the viral LTR and the adaptors from previous digested DNA using nested PCR. The hDNA-vDNA fragments generated with this method ranged from 100 to 2,000 bp in length, and contained the proviral long terminal repeat (LTR), the flanking genomic DNA and a linker adaptor. Highly purified hDNA-vDNA fragments were collected from nested PCR products using PCR clean-up kit (Cat#740609.250, Takara). 100ng of highly purified DNA were used to prepare dual-indexed paired-end sequencing libraries according to the Nextera™ DNA Flex Library Prep workflow (Illumina). Sixteen libraries were pooled together and were sequenced on a Miseq platform using a Miseq Reagent Kit v3 (600-cycle, Cat#MS-102-3003, Illumina). As a result of this procedure, the sequencing reads contain not only the genomic fragment needed for IS identification, but also viral and barcode sequences which was trimmed out (fastqc and trim-galore software) before alignment to the reference genome (bowtie2 and samtools software). Finally, aligned sequencing reads were annotated (deeptools/IGV/seqmonk software) to yield the final list of annotated viral integrated sites.

### Calculation of vector copy number

A Bio-Rad laboratories Auto DG QX200™ ddPCR system was used for detection of vector copy number of CAR T-cell products (bulk cells, not CAR^+^ T cells) and verification of integration sites in host genome. Detailed protocol was described in our previous publication [28].

### Transcriptome library preparation and sequencing

Total RNA was isolated from CAR T-cell products (transduced and non-transduced) using miRNeasy Mini Kit (Cat#217,084, Qiagen). Concentration and Quality were measured by Nanodrop 8000 (Thermo Fisher Scientific) and 2100 Bioanalyzer (Agilent). DNA libraries were performed using TruSeq Stranded Total RNA kit (Cat#20,020,598, Illumina) according to its protocol and sequenced on Illumina Nextseq 550 platform.

### Transcriptome data analysis

Raw fastq files were filtered by FastQC for quality control and processed with Trimmomatic to exclude adapter sequences and low-quality reads. Filtered reads were aligned against the human reference genome (GENCODE hg38) using STAR aligner. Gene expression level is quantified using subread (featureCounts). Differential expression analysis is performed using the limma package in Rstudio with custom scripts. Wald’s test was used to calculate the adjusted *p*-value or significance that a gene is differentially expressed. Genes with | FoldChange | >=2 and adj.P.value < 0.05 were considered significantly expressed. Differential alternative splicing transcripts were quantified using the rMATS software in a STAR output bam file. To find statistically differential splicing events, the threshold (FDR < 0.05, and ΔPSI ≥ 0.2) was executed. Student’s t-Test was used to calculate p-value when compared numbers of differentiate alternative splicing transcripts in lentiviral products and γ-retroviral products. p > = 0.05 represents no statistical significance. rmats2sashimiplot program was used to produce a sashimiplot visualization of rMATS output.

### Gene ontology analysis

Using the R package, clusterProfiler (version 3.0.4) [29], gene ontology (GO) analysis was performed on the dataset. GO analysis varies from GSEA as it utilizes a different annotation set and accounts for gene length bias in detection of over/ under representation of genes. With the hg37 annotation set, we performed enrichment analysis on our set of differentially enriched genes. We utilized log2(FC) and the *p*-value to determine significant genes for this analysis. Then we determined which GO terms were over or under-represented and visualized the data. We grouped the GO terms by biological process (BP), and selected the significant, over-represented terms. Adjusted *p*-value was calculated by the built-in function using clusterProfiler package in Rstudio.

### Principal component analysis

Principal component analysis (PCA) was conducted using factoextra package in R environment (version 3.6.1). Custom code was uploaded into GitHub public website as shown in our previous publication [30].

### Statistical analysis

All statistical analysis was performed with GraphPad Prism software and related R package. A *p*-value less than 0.05 was considered significant. Use of other statistic tests is indicated in each figure legend.

## Results

### Detection of viral vector integration sites using the VISA pipeline

**Fig. 1:**
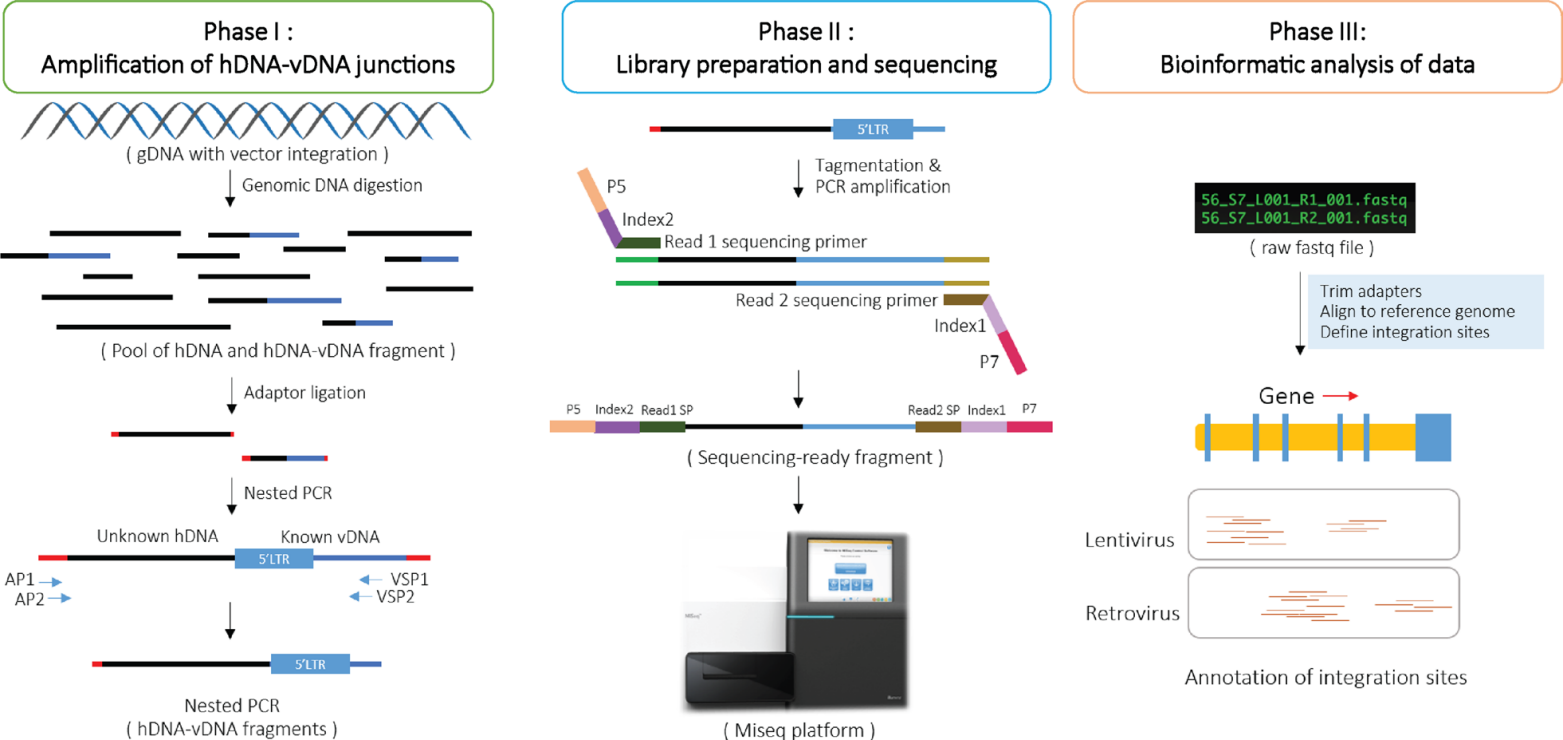
Overview of viral integration site analysis (VISA) pipeline. A simplified diagram of the viral integration site analysis pipeline. The process starts with genomic DNA extracted from CAR T-cell products. All details are described in Materials and Methods

To detect viral insertion sites in CAR T-cell products, we modified a vector integration sites analysis pipeline based on previous reports, which involves three phases (Fig. 1). Phase І is to construct pools of adaptor-ligated hDNA-vDNA fragments (chimeric DNA containing human genomic DNA sequences and viral vector sequences). In this phase, genomic DNA is extracted from transduced CAR T-cell products, digested with restriction enzyme DraI, end-paired, and ligated with GenomeWalker adaptors (supplied from the manufacturer) onto the ends of the digested DNA. Then, a nested PCR reaction was conducted with PCR primers designed to enrich the hDNA-vDNA fragments from these digested DNA. In phase II, the nested PCR products were used as the input to prepare DNA libraries for sequencing. Briefly, highly purified DNA was collected from the nested PCR products using a PCR clean-up kit. 100ng highly purified DNA was used to prepare dual-indexed paired-end sequencing libraries according to the NexteraTM DNA Flex Library Prep workflow (Illumina). Sixteen libraries were pooled together and analyzed by sequencing on the Miseq platform using a Miseq Reagent Kit v3 (600-cycle, Cat#MS-102-3003, Illumina). Finally, in phase III, the raw sequencing reads (fastq format) went through a bioinformatic workflow (Additional file 1: Fig. S1A) to define a set of viral vector integration sites in each CAR T-cell product. The workflow included quality control (fastqc), trim adaptor (trim-galore), alignment (bowtie2/samtools), and annotation (deeptools/IGV/seqmonk). To test the VISA pipeline, we used the Control Human Genomic DNA, which is offered in the Retro-X/Lenti-X Integration Site Analysis Kit (Cat#631,467, Cat#631,263, Takara) and has known integration sites at 17q21 upstream of the RSAD1 gene (γ-retrovirus) and at 2p13 within the TIA-1 gene (Lentivirus). As shown in Additional file 1: Fig. S1B, we observed the expected integration events at the indicated position. Thus, we used this pipeline to explore the viral integration sites in clinical CAR T-cell products prior to infusion.

### Preferential integration patterns of γ-retroviral and lentiviral vectors

**Fig. 2:**
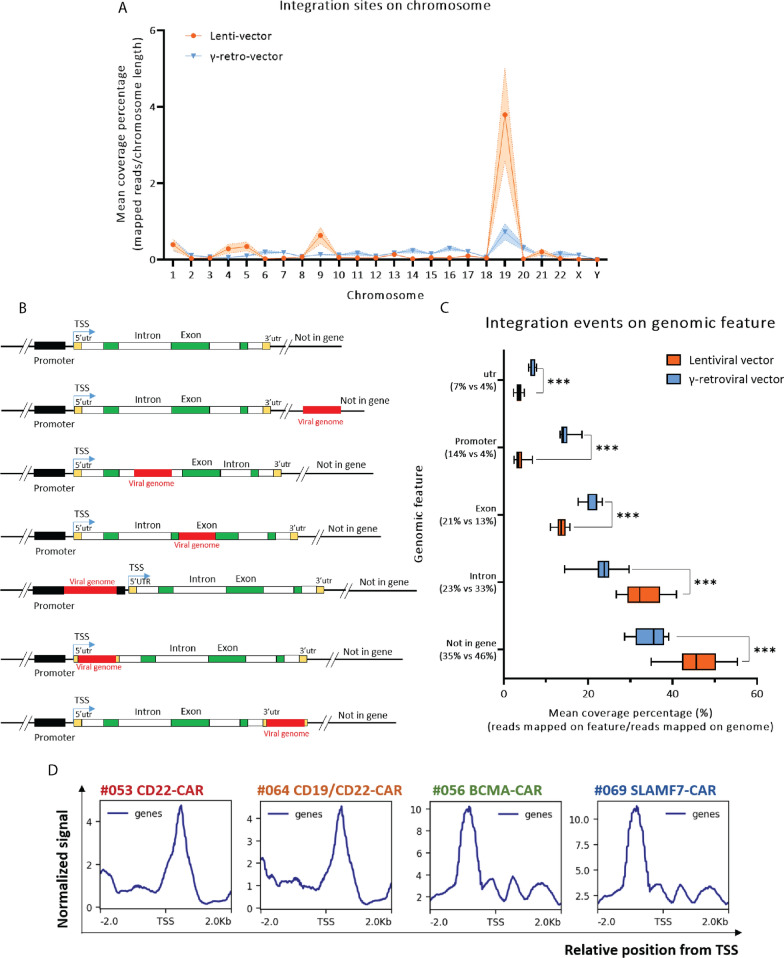
Genome-wide integration patterns of γ-retroviral and lentiviral vectors.**A** The percentage of viral integration sites on each human chromosome in CAR T-cells produced with lentiviral and γ-retroviral vectors is shown. **B** A scheme of expected integration sites in different genomic features. The viral genome is labeled as red rectangles. **C** The bar chart depicts the percentage of integration sites found in the intron, exon, promoter, untranslated region (utr, which includes 3’utr and 5’utr), and not in the gene region in all products. The x-axis represents mean coverage percentage (%). The y-axis represents each genomic feature. The number under each genomic feature in y-axis represents the mean coverage number of all samples in γ-retroviral vector group and lentiviral vector group. Data in boxplot were analyzed by using two-tailed unpaired Student’s t-Test. ***p < 0.001. **D** Representative patterns from 4 different CAR T-cell products showing the relative position of viral integration from the transcription start site (TSS). #053 CD22-CAR and #064 CD19/22-CAR were made with lentiviral vectors. #056 BCMA-CAR and #069 SLAMF7-CAR were made with γ-retroviral vectors. The x-axis indicates the relative position from transcription start site. The y-axis represents normalized signal

We analyzed CAR T-cell and TCR T-cell samples in order to identify vector integration sites (IS) across the whole genome. The analysis included 57 CAR T-cell products targeting different antigens manufactured with lentiviral vectors, 24 manufactured with γ-retroviral vectors (Additional file 6: Table S1). Sequencing fastq files were mapped onto the human reference genome to define the overall distribution pattern of the insertion sites in all chromosomes and in different genomic structures. As shown in Fig. 2A, for all samples, the greatest enrichment of insertion sites occurred in Chromosome 19. Neither vector nor CAR type appeared to influence the pattern of greater insertion into Chromosome 19 (Fig. 2A and Additional file 2: Fig. S2A and S2B). This is consistent with other studies which have found that viral vectors prefer integrating into gene-dense regions [31, 32] and chromosome 19 has the highest gene density of all chromosomes (Additional file 2: Fig. S2C). We also found that lentiviral vectors had a higher level of enrichment in Chromosome 19 than γ-retroviral vectors (nearly 3.5 times, Fig. 2A).

Next, we explored the proportion of insertion events occurring at each genomic structure. Possible vector insertion sites relative to gene structures such as promoters, introns, exons and untranslated regions (utr) are shown in Fig. 2B. When the promoter, utr, and exon regions were analyzed, we found that γ-retroviral vectors were more likely to insert into these regions compared with lentiviral vectors, while lentiviral vector integration sites were more likely to occur in intron and intergenic regions compared with γ-retroviral vectors (Fig. 2C). More specifically, 54% of lentiviral vector and 65% γ-retroviral integration sites were located in the region of a specific gene. Of these insertions, 33% vs. 23% occurred in introns for lentivirus and retrovirus vectors respectively; 13% vs. 21% in exons; 4% vs. 14% in promoters; and 4% vs. 7% in utr (Fig. 2C). The results of analysis of each CAR T-cell product showed a similar pattern for all products made with each unique vector (Additional file 2: Fig. S2D). Furthermore, closer evaluation of the position of integration events occurring in the promoter region (2 kb upstream and 500 bp downstream of transcription start site), revealed an obviously different pattern of γ-retroviral and lentiviral vectors integration at transcription start site (TSS). Lentiviral vectors mainly integrated into the gene 500 bp downstream of TSS, while γ-retroviral vector insertion sites were enriched at sites 1 kb upstream of TSS (Fig. 2D). Above all, although much more integration of lentiviral vectors occurred in gene-dense regions, most often the lentiviral vectors integrated into the introns. Given the respective function of each genomic feature in gene expression, our results suggest lentiviral vectors likely change cellular gene expression mainly by affecting post-transcriptional modification, such as altering alternative splicing type, while, γ-retroviral vectors likely affect cellular transcriptome mainly by integrating into gene promoter region.

### Gene locus of γ-retroviral and lentiviral vector integration sites

**Fig. 3:**
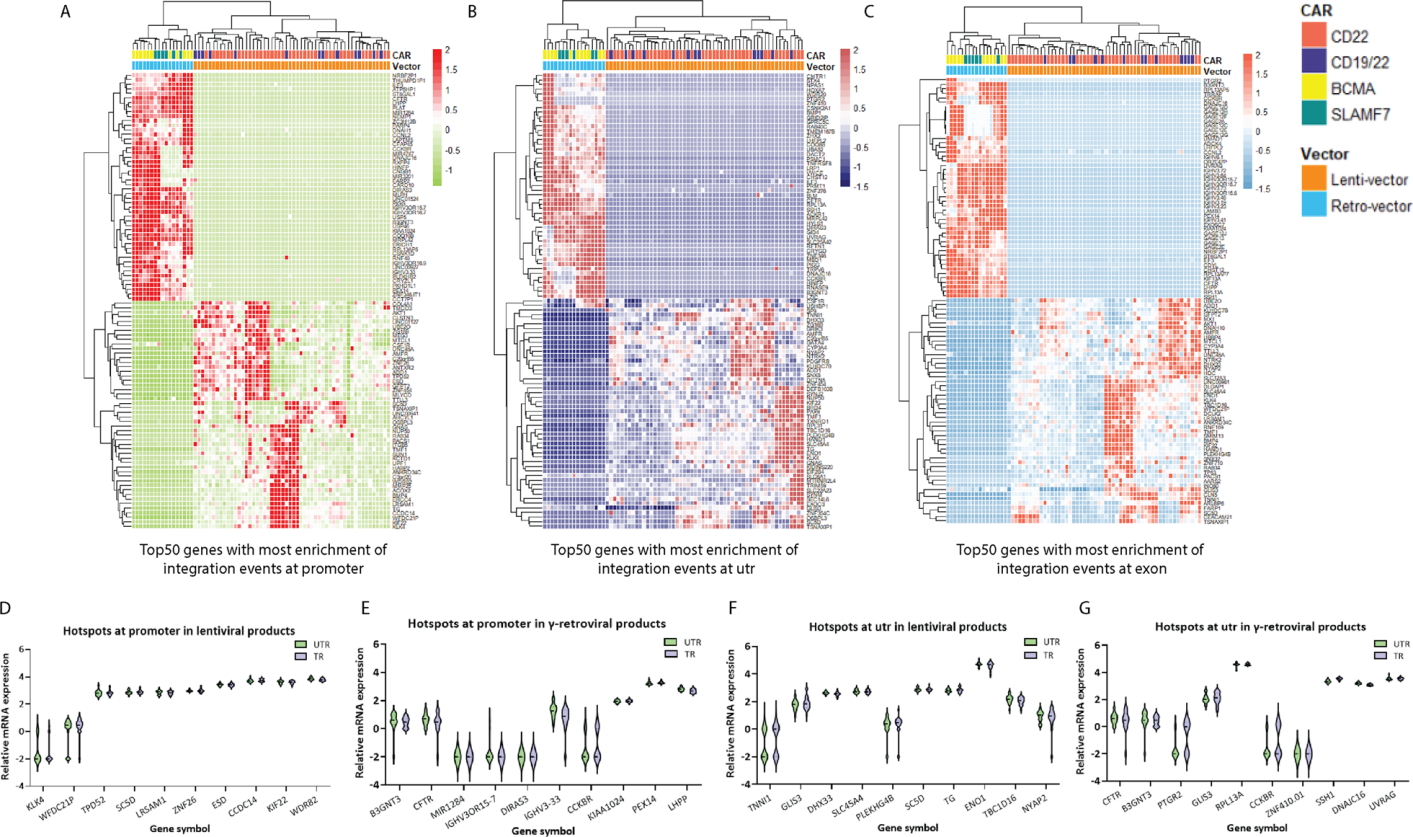
Hotspots of viral integration at gene loci and their mRNA expression. ** A**–**C** Unsupervised hierarchical clustering of normalized integration data by samples (columns) and genes (rows) are shown. Data normalization was scaled using the built-in R function (scale). Lentiviral and γ-retroviral vectors showed different integration hotspots. **A** Heatmap showing the top 50 genes with the most enrichment of integration events at the promoter (genes with integration events from higher enrichment to lower enrichment are marked from red to green). **B** This heatmap shows the top 50 genes with the most enrichment of integration sites at the untranslated region (genes with integration events from higher enrichment to lower enrichment are marked from dark red to purple). **C** The 50 genes with the most enrichment in the exon region are shown (genes with integration events from higher enrichment to lower enrichment are marked from bittersweet to sky-blue). The bars at the top of each heatmap indicate CAR (CD22: bittersweet; CD19/22: vivid violet; BCMA: yellow; SLAMF7: jungle green) and viral vector type (lenti-vector: orange; Retro-vector: light blue). **D–G**) Gene expression showed no differences among transduced (TR) vs. non-transduced (UTR) cells for hot spot genes that integrated into the promoter (**D**, **E**) or the untranslated region (utr, **F** and **G**). Differential expression analysis was performed using the built-in function from limma package in Rstudio with custom scripts. Wald’s test was used to calculate the adjusted *p*-value or significance that a gene is differentially expressed. Genes with | FoldChange | >=2 and adj.P.value < 0.05 were considered significantly expressed

To determine if some genes were more likely to contain viral vector integration sites, we identified genes in all CAR T-cell products with integration events at the promoters, untranslated regions, and exons. The top 50 genes which were most likely to have an insertion event at one of these three regions for CAR T-cell products produced with lentiviral and γ-retroviral vectors is summarized in Fig. 3. As expected, the two types of vectors had different integration hotspots. For example, the NRBF2P1 gene promoter had a vector integration site in all 17 CAR T-cell products made with γ-retroviral vectors, while this same promoter has no integration events in any of the 54 CAR T-cell products made with lentiviral vectors (Fig. 3A). In contrast, the untranslated region of TNNI1 had an insertional site in most CAR T-cells produced with lentiviral vectors, but this region had no integration events in any of the CAR T-cells manufactured with γ-retroviral vectors (Fig. 3B). Similarly, the integration sites for CAR T-cell products manufactured with γ-retroviral vectors were more likely to be in exons than for CAR T-cell products manufactured with lentiviral vectors (Fig. 3C). These integration hotspots further highlight the differences in integration patterns among lentiviral and γ-retroviral vectors. Strikingly, we found that the expression of these “hotspot” genes was not affected even if the integration site was in the promoter and utr region (Fig. 3D–G).

### Effects of viral vector integration events at gene promoter and untranslated region on gene expression

**Fig. 4:**
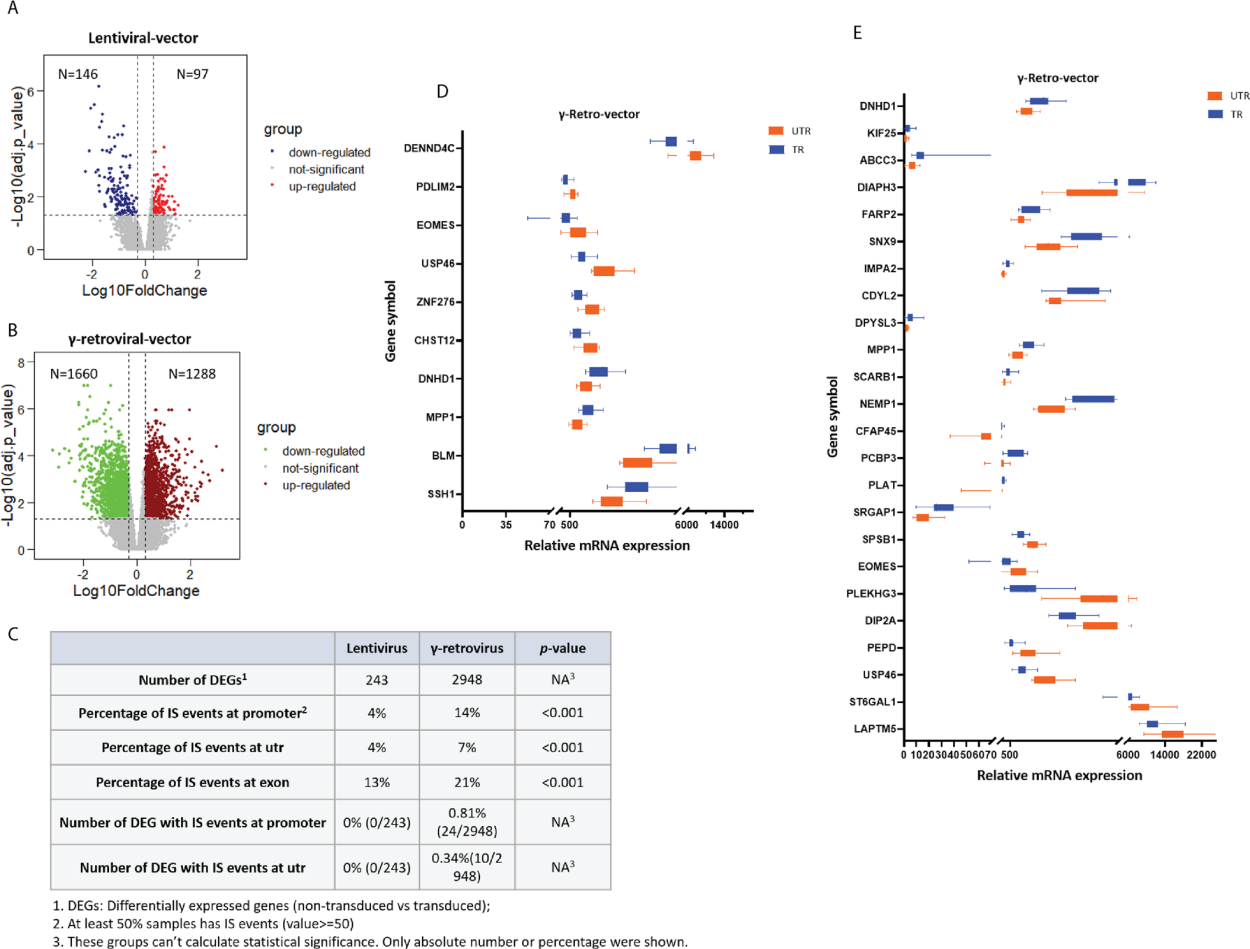
Effect of viral vector integration into the gene promoter and untranslated region. ** A**, **B** Volcano plots show the differentially expressed genes between control non-transduced cells vs. CAR T-cells transduced for lentiviral (**A**) and γ-retroviral vectors (**B**). The number of DEGs were indicated in the volcano plot. Significantly downregulated genes are highlighted in blue (**A**) or green (**B**) and upregulated genes are highlighted in red (**A**) or brown (**B**). Differential expression analysis was performed using the built-in function from limma package in Rstudio with custom scripts. Wald’s test was used to calculate the adjusted *p*-value or significance that a gene is differentially expressed. Genes with | FoldChange | >=2 and adj.P.value < 0.05 were considered significantly expressed. **C** The number of DEGs and integration events occurring at promoter and untranslated region of these DEGs is summarized. DEGs indicates differentially expressed genes; IS events indicates integration site events; and utr, untranslated region. NA means no value is available in the *p*-value column. Percentage = sequencing reads mapped on each feature/sequencing reads mapped on genome. The boxplots show the expression of 34 genes with integration events at untranslated region (**D**) and promoter (**E**) and whose expression differed in control non-transduced vs. transduced CAR T-cells

Given the important transcription regulatory functions of the promoter and utr region, we evaluated their effect on global gene expression by measuring mRNA levels using RNA-sequencing. We evaluated all CAR T-cell products and paired non-transduced cells and identified the genes that were differentially expressed among the two groups (Additional file 7: Table S2). As shown in Fig. 4A and B, more differentially expressed genes (DEGs) were identified in CAR T-cell products manufactured with γ-retroviral vectors than those manufactured with lentiviral vectors.

Next, we identified all DEGs with insertional events at the promoter and untranslated region. We found only 34 or 1.15% of all DEGs had integration at these regions. Among CAR T-cell produced with lentiviral vectors, none were identified with insertional events at the promoter and untranslated region. In contrast, for CAR T-cells produced with γ-retroviral vectors, 0.81% and 0.34% of DEGs had integration at promoter and untranslated regions, respectively (Fig. 4C). The expression of these 34 genes expression is shown in Fig. 4D and E. Our results indicated integration events that occurred at promoter and utr regions have little impact on gene mRNA expression. These DEGs may be the indirect consequences of integration events that occurred at exon regions which induced abnormal transcriptional factors by changing amino acid sequence. We did find that the percentage of integration events in exons were greater in γ-retroviral products than in lentiviral products (Fig. 4C). In addition, we found that there was some overlap in genes whose expression was changed by the two types of vectors (Additional file 3: Fig. S3A). Interestingly, we found that most of the overlapping DEGs encoded proteins localized to the cell membrane and were involved in the regulation of innate immune response (Additional file 3: Fig. S3B and 3C). This suggested that these DEGs may act as sensors at the membrane to perceive virus infection and may be involved in the regulation of innate immune response to virus infection.

### Effects of insertional events and alternative gene splicing

**Fig. 5:**
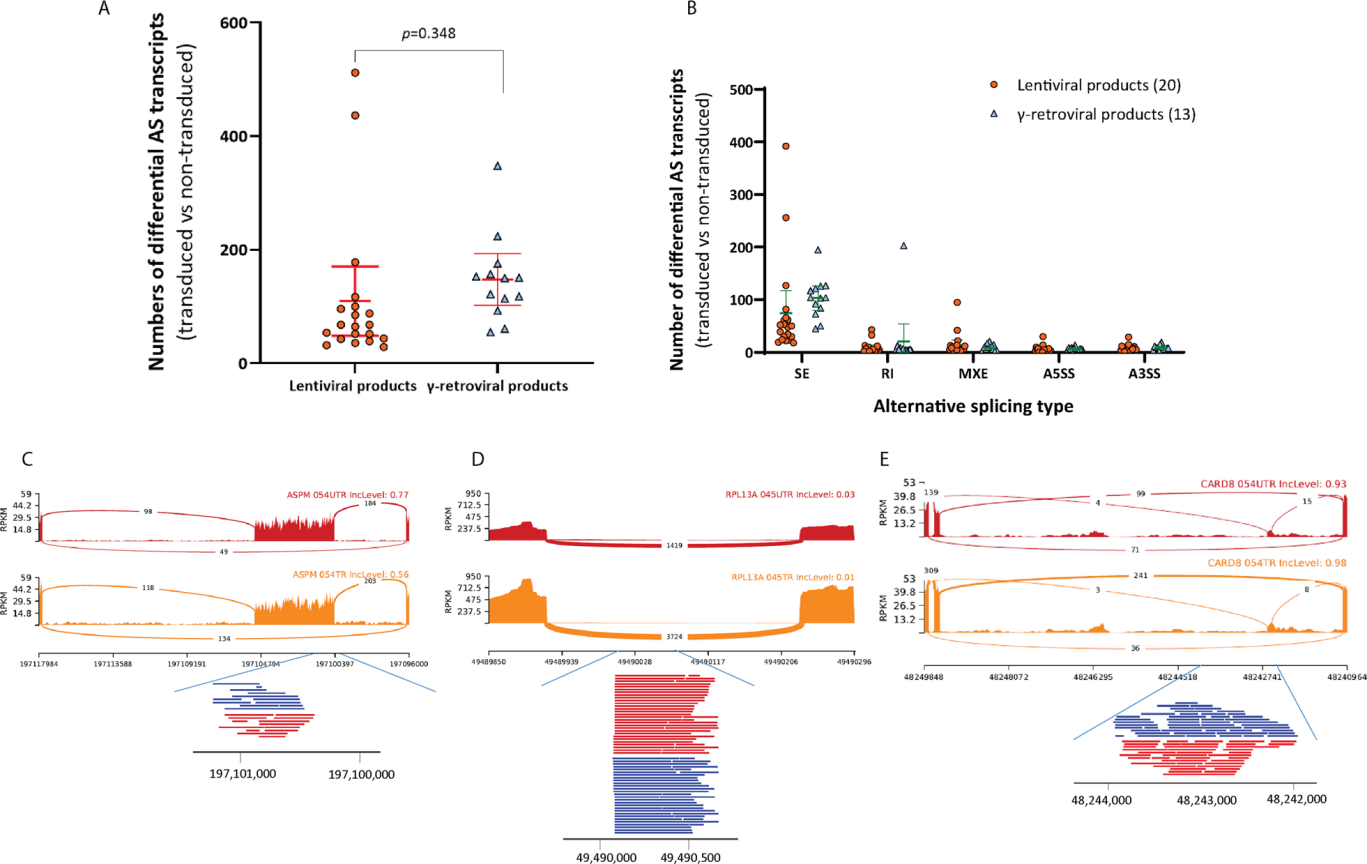
Integration events occurring in exon/intron regions affect alternative splicing transcripts. ** A** The scatter plot shows the number of differential alternative splicing transcripts based on the analysis of control non-transduced vs all transduced CAR T-cell products. The median and 25th-75th percentiles are indicated in lentiviral and γ-retroviral products. Student’s t-Test was used to calculate *p*-value. *p* > = 0.05 represents no statistical significance. AS transcripts, alternative splicing transcripts. **B** All differential alternative splicing transcripts are summarized according to different alternative splicing type. SE, skipped exon; RI, retained intron; MXE, mutually exclusive exon; A5SS, alternative 5’ splice sites, A3SS, alternative 3’ splice sites. **C–E** The Sashimi plots show integration events occurring at alternative exon/intron region and impaired skipped exon (**C**), retained intron (**D**), and mutually exclusive exon (**E**) process based on RNA-seq analysis. The height of the peaks shows exon coverage, the number in the red (orange) lines show the number of splicing reads. The black scheme under the plot shows the position of the gene in genome. Integration events are showed with blue/red horizonal line under alternative exon or intron position

In addition to promoter and untranslated region, we also explored the effect of integration events in exons and introns, which are also key components of post-transcriptional regulation, especially in alternative splicing. Alternative splicing (AS) is one of the most powerful means of modulating gene expression and increasing protein diversity. It has been reported that insertion events from viral vectors can change the nature of alternative gene splicing [33, 34]. First, we explored all differentially expressed alternative splicing transcripts in transduced CAR T-cell products (compared with non-transduced control cells) based on RNA-sequencing data. Specifically, we looked for skipped exons (SE), retained introns (RI), mutually exclusive exons (MXE), alternative 5’ splice sites (A5SS), and alternative 3’ splice sites (A3SS). As shown in Fig. 5A, we identified many differential transcripts with AS. Among these, SE events generated most of the differential transcripts with AS (Fig. 5B). Moreover, there was a trend that γ-retroviral vectors caused more differential AS transcripts than lentiviral vectors, but the *p* value was not significant (*p* = 0.348).

Then, we checked whether vector integration occurred at these AS sites in each CAR T-cell product. Though most alternative splicing sites had no integration events, we did find several interesting vector integration sites in some products (Additional file 8: Table S3). Figure 5C–E depicts the detailed situation where vector integration events in exons and introns resulted in alternative splicing transcripts. For example, in product #054, there was a differential alternative splicing transcript due to a skipped exon at the 18th exon of the ASPM gene. Meanwhile, there were vector integration events inside the 18th exon of the ASPM gene (Fig. 5C). Similarly, differential expression of transcripts resulting from retained introns or mutually exclusive exons also showed vector integration events at these alternative introns and exons in #045 and #054 (Fig. 5D, E).

Thus, these results indicated the possibility that viral vector integration events in exon and intron regions can regulate mRNA transcripts through affecting alternative splicing. These events were, however, of low frequency and high randomness and we found in only 8 differential AS transcripts that were associated with integration sites and none of the 8 AS were found in two or more products. On the other hand, the results highlighted the necessity and importance to monitor integration events in each individual CAR T-cell product. Also, it could be important to go back to the product to check the viral integration when severe event occurs in therapy.

### Vector integration sites and CAR T-cell specificity, patient disease and patient gender among different CAR T-cells

**Fig. 6:**
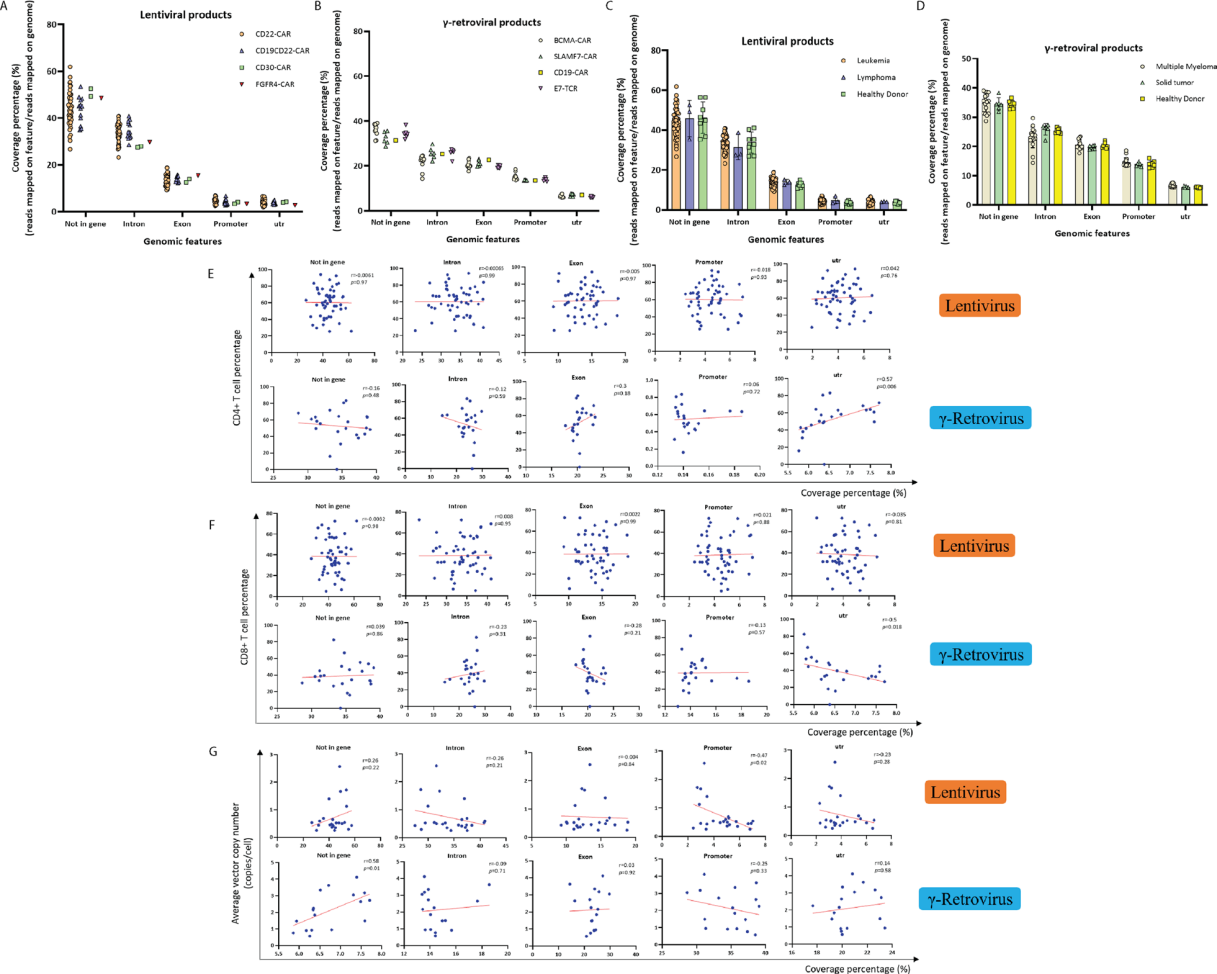
Factors associated with vector integration in CAR T-cell products. The percentage of integration events at genomic features based on CAR T-cell type in lentiviral (**A**) and γ-retroviral products (**B**), and based on CAR T-cells produced from patients with different diseases (**C**, **D**). The scatter plots show a correlation between percentage of integration at each genomic features and percentage of CD4^+^ T cells (**E**), percentage of CD8^+^ T cells (**F**), vector copy number (**G**) in CAR T-cells produced with the lentiviral and γ-retroviral vectors. The x-axis indicates coverage percentage (%). The y-axis represents CD4 + T cell percentage (**E**), CD8 + T cell percentage (**F**) and Average vector number (**G**). Pearson correlation coefficients (r) and two-tailed *p*-value were computed in the GraphPad software

Whether characteristics of CAR T-cell products have an impact on vector integration sites is unknown. To address this issue, we first compared the vector integration site profiles among CAR T-cells produced with different vectors. The CAR T-cells produced with the 4 different lentiviral vectors showed similar proportions of integration at each genomic feature as did the CAR T-cells produced with the 3 different γ-retroviral vectors (Fig. 6A, B). We also compared integration sites of CAR T-cells produced using γ-retroviral vectors with six TCR engineered T-cell products targeting HPV-16 E7 oncoprotein which were also produced with γ-retroviral vectors. We found no difference in coverage percentage between CAR T-cell products and these TCR T-cell products (Fig. 6B). These results show that γ-retroviral vectors encoding different receptors had similar integration site profiles as did lentiviral vectors.

In addition, we found that the viral vector integration patterns were not host- or gender-specific. A similar distribution percentage can be observed in CAR T and TCR-engineered cells derived from patients of different gender and with different diseases (Fig. 6C and D, Additional file 4: Fig. S4A and B). These results suggest that CAR T-cell vectors and recipient’s disease and gender have no influence on the preference of integration events for specific genomic features.

### Vector integration sites and CAR T-cell product CD4 and CD8 cell composition, transduction efficiency and vector copy number

We explored whether the proportion of CD4^+^ and CD8^+^ T-cells in CAR T-cell products affects vector integration patterns. In addition, we assessed the effect of CAR T-cell vector transduction efficiency (TE), and vector copy number (VCN) on vector integration. For products made with γ-retroviral vectors, the proportion of CD4^+^ and CD8^+^ T cells in the CAR T-cell product was associated with vector integration in untranslated regions, but not for CAR T-cell products manufactured with lentiviral vectors (Fig. 6E, F). The percentage of γ-retroviral vectors integrating into untranslated regions was positively correlated with CD4^+^ T cell (r = 0.57, *p* = 0.006), while it was negatively correlated with CD8^+^ T cell (r =− 0.5, *p* = 0.018). There was a trend among both lentiviral and γ-retroviral CAR T-cell products that higher vector copy number was correlated with more integration at non-gene regions, and less integration in promoter regions (Fig. 6G). There was no statistically significant correlation between transduction efficiency and viral integration percentage coverage among all CAR T-cell products (Additional file 4: Fig. S4C). In summary, these data indicated the characteristics of CAR T-cells can affect viral integration site selection in untranslated regions in γ-retroviral products.

### Vector integration sites and clinical outcomes of CAR T-cell therapy

It has been reported that viral vector integration site distribution in CAR T-cells is linked to patient clinical outcomes [35]. We investigated the relationship between viral integration events and clinical outcomes in a subset of patients (n = 28) treated with CD22-CAR T-cells. Of these, 23 (82.1%) patients had clinical responses (complete remission) and 5 (17.9%) had no clinical response. Concerning toxicities: 4 (14.3%) developed high-grade cytokine release syndrome [36] (CRS ≥ grade 3 as per Lee criteria), and 13 (46.4%) developed hemophagocytic lymphohistiocytosis-like manifestations (carHLH [37]).

**Fig. 7:**
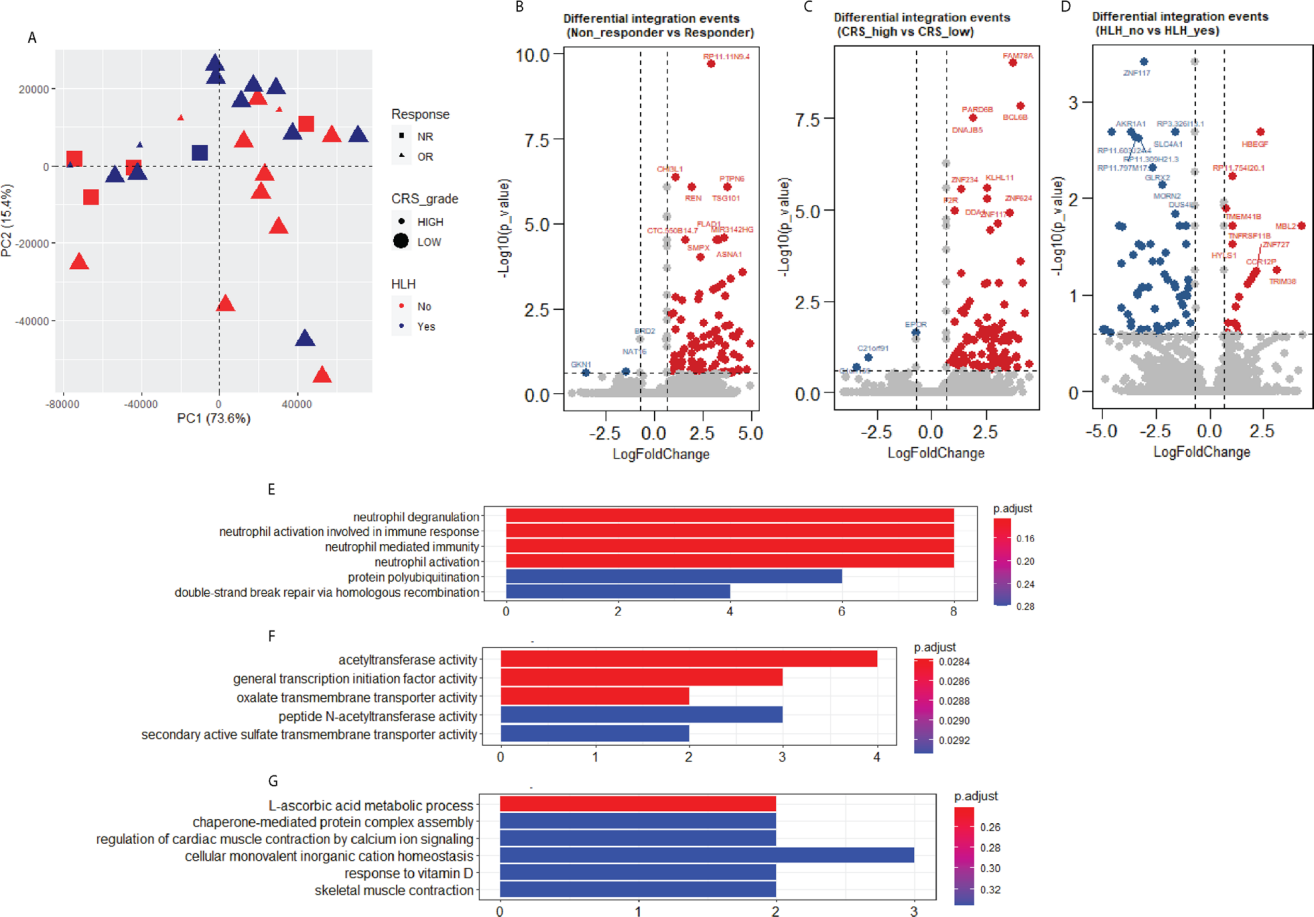
Vector integration sites and clinical outcomes of CAR T-cell therapy. **A** PCA (principal component analysis) plot using normalized gene integration data does not show distinct clustering of non-responder vs. responder, high grade CRS vs. low grade CRS, or HLH + vs. HLH−. OR: Objective response; NR: Non-response; CRS: Cytokine release syndrome; HLH: Hemophagocytic lymphohistiocytosis. B, C and D) Volcano plot of differential gene integration events comparing in non-responder and responder (**B**), CRS_high and CRS_low (**C**), HLH_no and HLH_yes (**D**). The red dots represent genes with higher enrichment of viral integration in Non-responder group, CRS_high group, and HLH_no group. The blue dots represent genes with lower enrichment of viral integration in Non-responder group, CRS_high group, and HLH_no group. The grey dots are genes without statistical significance.** E**–**G** Genes with more integration events in non-responders (**E**), more integration events in patients with low grade CRS (**F**), and more integration events in patients with HLH (**G**) were analyzed using gene ontology (GO) analysis. The y-axis indicates the different biological processes and the x-axis represents genes count involved in each biological process. Adjusted *p*-value was determined with the built-in function using clusterProfiler package in Rstudio

The amount of viral vector integration in specific gene regions was used to perform principal component analysis (PCA) and the result did not reveal a clear separation of patients with different clinical outcomes (Fig. 7A). Also, vector integration into each specific genomic feature showed no difference among CAR T-cell products given to patients with a clinical response to those without a response (Additional file 5: Fig. S5A–C).

However, clinical outcome was associated with integration into specific genes (Fig. 7B–D and Additional file 9: Table S4). A total of 109, 111, 103 genes showed differential integration among clinical response, CRS, and HLH groups (Fig. 7B–D and Additional file 9: Table S4). However, none of these genes showed any disrupted expression or over-expression (data not shown). We speculated that these integration events were in the gene body and may have disrupted the amino acid sequence and generated abnormal proteins, instead of affecting mRNA expression. Gene ontology was performed on these genes to identify enriched pathways. GO analysis of genes with more integration events in non-responders revealed that most of these genes are enriched in neutrophil activation and associated neutrophil immune response pathways (Fig. 7E). Genes with more integration events in CAR T-cell products from patients experiencing high-grade CRS were most commonly found in pathways involved in acetyltransferase/transmembrane transporter activity (Fig. 7F). Genes with more viral integrations in CAR T-cells associated with those patients who developed HLH-like manifestations showed a small association with metabolic process pathways (Fig. 7G). In summary, our results indicate that individuals with different clinical outcomes showed a group of genes with differential enrichment of integration events. These genes that affected patient outcomes may have affected CAR T-cell function by interrupting amino acid sequences and generating abnormal proteins, rather than by affecting their mRNA expression.

## Discussion

Though retroviral vectors are widely used in manufacturing CAR T-cell products for immunotherapy, little is known about integration sites of γ-retroviral and lentiviral vectors in CAR T-cells. In this study, we effectively modified a reproducible pipeline to systematically monitor CAR T-cell viral vector integration sites, investigate insertional effects on host gene expression and to explore potential association between integration events with clinical outcomes.

We found both lentiviral and γ-retroviral vectors have their own specific integration patterns and integration hotspot loci. We found that γ-retroviral vectors were more likely to insert into promoter, utr, and exon regions compared with lentiviral vectors, while lentiviral vector integration sites were more likely to occur in intron and intergenic regions, when compared with γ-retroviral vectors.

We found that integration events affected gene expression at the transcriptional and post-transcriptional level. These integration sites could be affected by the characteristics of γ-retroviral CAR T-cell products, such as the proportion of CD4^+^ and CD8^+^ T-cells. More importantly, some genes were identified in CD22 CAR T-cell products that showed differential viral vector integration based on clinical outcomes.

Factors used as predictive markers of clinical outcomes to CAR T-cell immunotherapy have been reported in recent years. The nature of T-cell subsets and the expression of immune checkpoints expressed by T-cells before CAR T-cell manufacturing are factors described as influencing the efficiency of CAR T-cell products [38–41]. CAR T-cell expansion and persistence have also been described as two potential markers of clinical response [42, 43]. We found that clinical outcomes were associated with CAR T-cell differential vector integration events. Interestingly, in non-responders, more integration events were found in genes mainly involved in neutrophil activation, which may mediate immune suppression activity [44, 45]. These results suggest that further study of differential integration events could reveal additional useful predictive markers for clinical outcomes, recognizing the limitation, however, that these analyses are rarely done in real-time and are often assessed after patients are treated.

Preferential integration has been previously reported in HIV/MLV infected cell lines and these studies found that lentiviral vector integrates primarily within bodies of actively transcribed genes and γ-retroviral vector preferentially target active promoters [46, 47]. Given that viral vectors used in CAR T-cell manufacturing are genetically engineered and some contents differ when compared with the original viral sequence, it’s essential to explore vector integration patterns in CAR T-cell products. We found some difference in the distribution of integration in genomic structures in CAR T-cell products. Our results showed that lentiviral vectors mainly inserted into non-coding regions (intergenic and intron) and γ-retroviral vectors have a higher integration percentage in promoter, exon and untranslated regions when compared with lentiviral vectors.

To date, the question as to whether viral integration occurs randomly or not is still a matter of debate. The general viewpoint is that integration is semi-random which means viral integration at each genomic feature is not random, but random at each gene locus [48, 49]. We found that some gene loci are hotspots for viral integration in CAR T-cells which indicates that these were non-random integration events. Interestingly, these hotspot genes differed significantly between lentiviral and γ-retroviral vectors. None of the previously published studies reported hotspots of viral integration at gene loci. Our data provides evidence that viral integration at gene loci are only semi-random. As to the question of why these gene loci were selected as hotspots, the main factors influencing integration is the sequence of the viral vector and genomic factors such as gene accessibility, GC content, and epigenetic modification.

We found that lentiviral and γ-retroviral vectors had their own distinct integration pattern and hotspots gene loci. The different pattern also reflected on transcriptional differences. CAR T-cell products made with γ-retroviral vector showed more differentially expressed genes and had more viral integration events at promoter and untranslated regions. Among these DEGs, we found that only 1% of CAR T-cell products had integration events at their promoter and utr region in those manufactured with γ-retroviral vector, while there were no integration events in these regions of CAR T-cell products manufactured with lentiviral vectors. Furthermore, we found that among the differentially expressed genes there were both up-regulated and down-regulated genes in the CAR T-cell manufactured with γ-retroviral (Fig. S3D and E). We speculate that the nature of the changes in gene expression depend on the relative orientation between integrated vector and gene promoter. Gene expression was enhanced when the orientation was the same as the cellular gene and reduced when the gene was in the opposing direction. In addition, we also showed that integration events could affect mRNA transcripts at post-transcriptional level by impairing alternative splicing when integration events altered exons and introns. Given the percentage of DEGs with viral integration events in gene loci, our findings indicated that most of integration events have little direct impact on gene expression. Most of DEGs without integration in gene loci could be the result of indirect regulation of integration events, which may insert into regulatory elements and impaired their regulatory role for gene expression [11, 50].

Our study was limited in that we were unable to track the CAR T-cells post-infusion to determine if the cells expanded clonally due to we are unable to get patient samples after CAR T-cell infusion. A recent study has described the dominance of a single infused CAR T-cell clone in a single patient that was associated with integration into the TET2 gene [6]. Another study found integration into CBL gene generated detectable expansion of CAR T-cell clones [7]. Future research could include the possibility of studying long-term impacts of viral integration events.

In summary, we comprehensively explored viral vector integration sites in pre-infusion CAR T-cell products based on a modified VISA pipeline and verified the semi-random nature of viral integration into the genome. We found that individuals with different clinical outcomes showed a group of genes with differential enrichment of integration events and the function of these genes may be disrupted by interrupting amino acid sequence and generate abnormal proteins, instead of affecting mRNA expression. Whether viral vector integration sites correlated with clinical outcomes warrants further study. Most importantly, we found differences in integration patterns, insertion hotspots and effects on gene expression vary between lentiviral and γ-retroviral vectors used in CAR T-cell products and established a foundation upon which we can conduct further analyses.

## Supplementary Information

**Additional file 1: Figure S1.** Bioinformatic workflow and positive control for VISA pipeline. Related to Figure 1.

**Additional file 2: Figure S2.** Viral integration sites on chromosomes in products with different CAR type and each individual. Related to Figure 2.

**Additional file 3: Figure S3.** Annotation of shared differentially expressed genes between lentiviral and γ-retroviral CAR T-cell products. Related to Figure 4.

**Additional file 4: Figure S4.** Percentage of integration events at each genomic features based on gender and transduction efficiency. Related to Figure 6.

**Additional file 5: Figure S5.** Percentage of integration sites at genomic features in each group with different clinical outcomes. Related to Figure 7.

**Additional file 6: Table S1.** Characteristics of CAR T-cell products.

**Additional file 7: Table S2.** Differentially expressed genes in non-transduced and transduced CAR T-cell products.

**Additional file 8: Table S3.** Differential alternative splicing transcripts with viral integration.

**Additional file 9: Table S4.** Differential integration sites at gene locus based on clinical outcomes.

## Data Availability

Patient-related data not included in the paper were generated as part of completed or ongoing clinical trials and may be subject to patient confidentiality. Therefore, these data may be restricted. With regard to sequencing data, raw fastq files will upload into public GEO datasets upon manuscript was accepted. Any other data that support the findings of the study are available from the corresponding author upon reasonable request.
