# Supplementary figures and images for: Genome-wide profiling of retroviral DNA integration and its effect on clinical pre-infusion CAR T-cell products
Source: J Transl Med. 2022 Nov 8;20:514. doi: 10.1186/s12967-022-03729-5 (PMC9644589; doi:10.1186/s12967-022-03729-5)

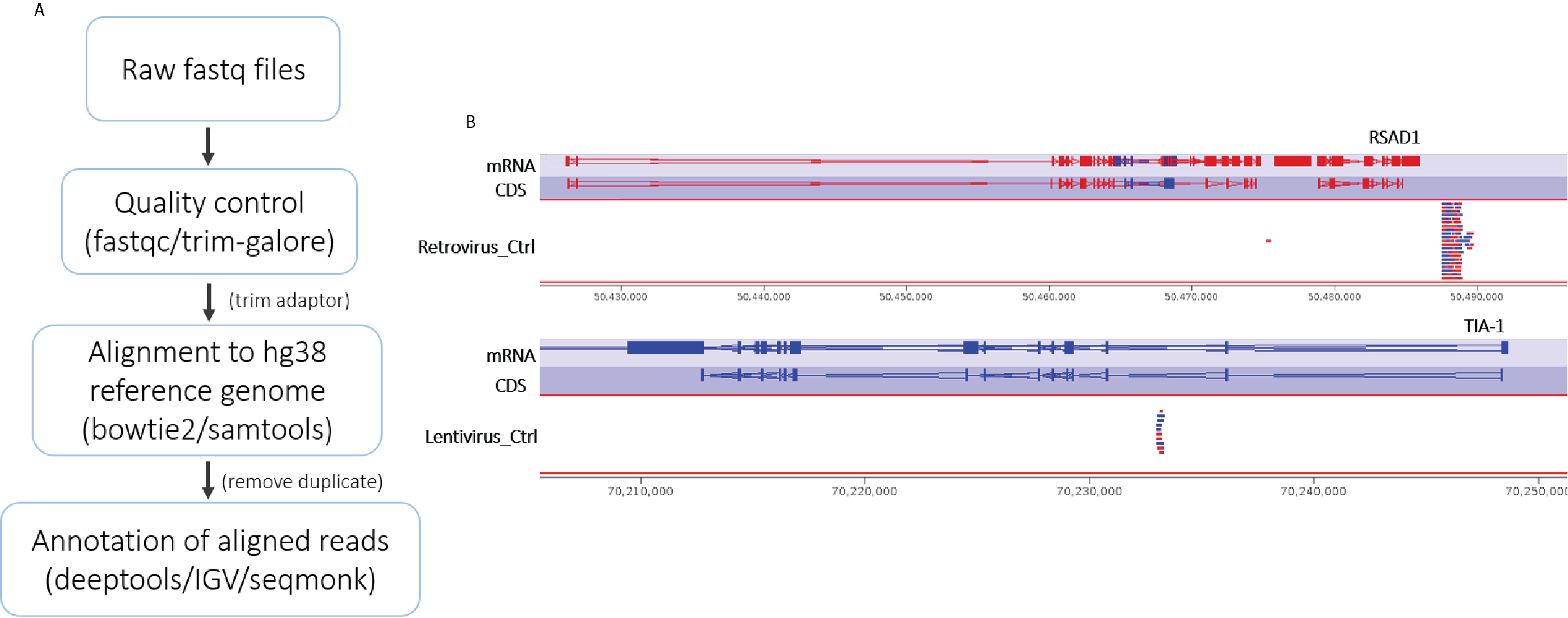

Supplement: Supplementary file 1 — Additional file 1: Figure S1. Bioinformatic workflow and positive control for VISA pipeline. Related to Figure 1. [file 12967_2022_3729_MOESM1_ESM.tif]

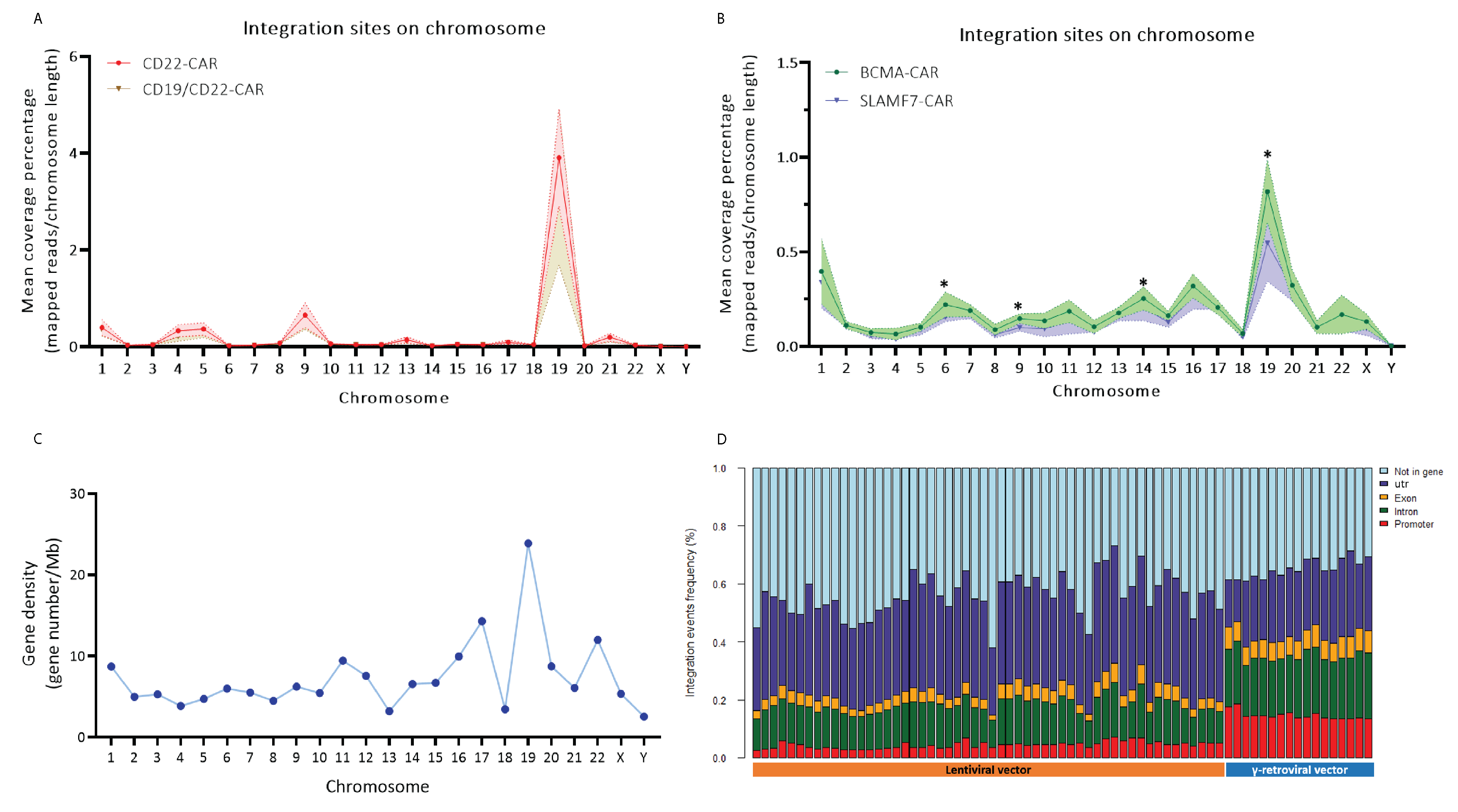

Supplement: Supplementary file 2 — Additional file 2: Figure S2. Viral integration sites on chromosomes in products with different CAR type and each individual. Related to Figure 2. [file 12967_2022_3729_MOESM2_ESM.tif]

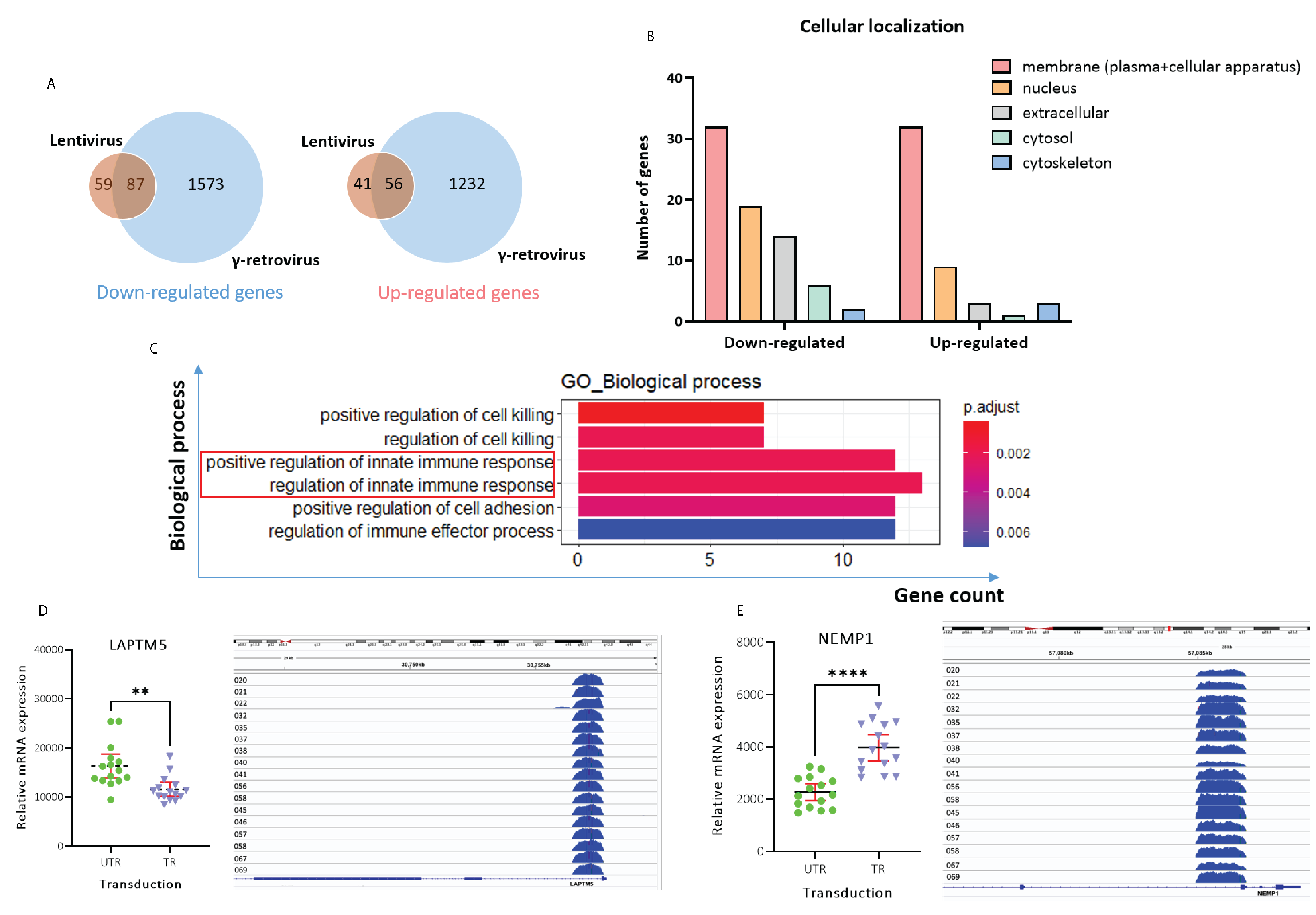

Supplement: Supplementary file 3 — Additional file 3: Figure S3. Annotation of shared differentially expressed genes between lentiviral and γ-retroviral CAR T-cell products. Related to Figure 4. [file 12967_2022_3729_MOESM3_ESM.tif]

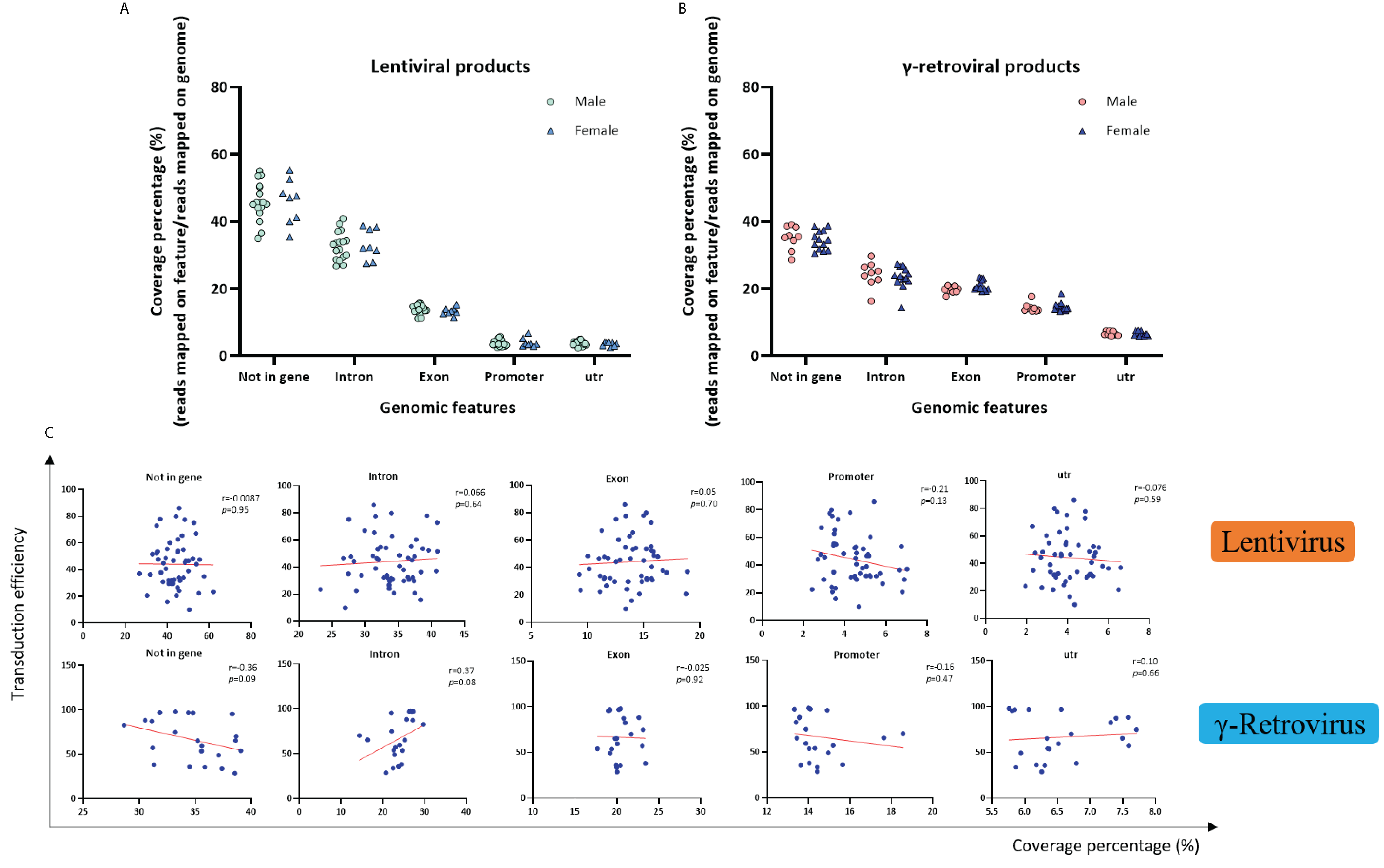

Supplement: Supplementary file 4 — Additional file 4: Figure S4. Percentage of integration events at each genomic features based on gender and transduction efficiency. Related to Figure 6. [file 12967_2022_3729_MOESM4_ESM.tif]

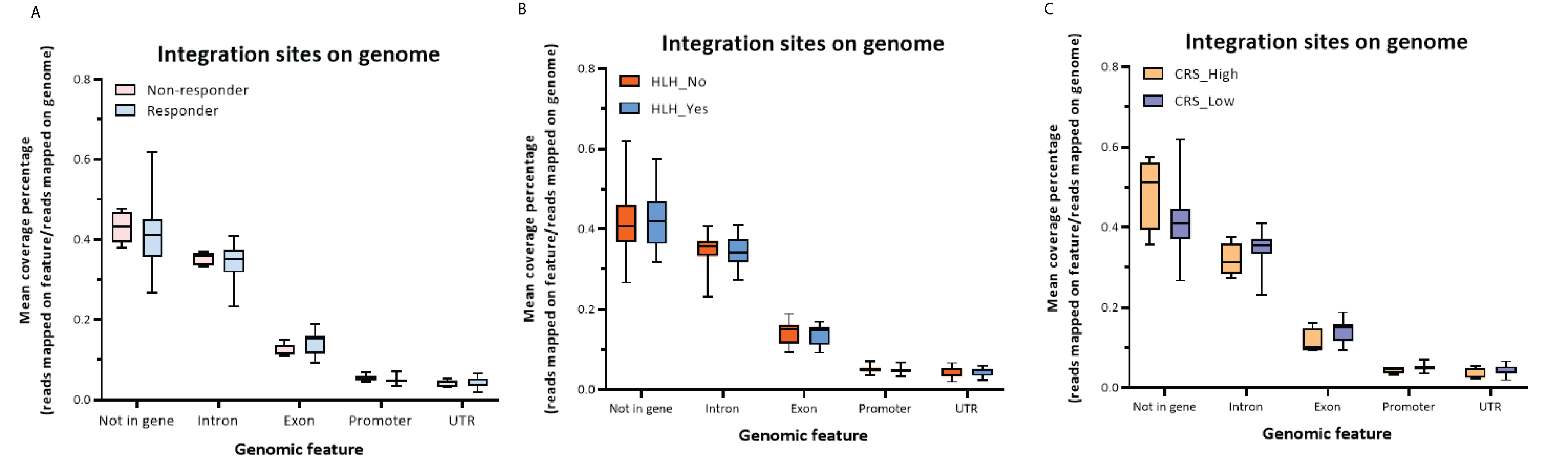

Supplement: Supplementary file 5 — Additional file 5: Figure S5. Percentage of integration sites at genomic features in each group with different clinical outcomes. Related to Figure 7. [file 12967_2022_3729_MOESM5_ESM.tif]
